# Interaction Between NOD2 and Smoking in the Pathogenesis of Crohn's Disease

**DOI:** 10.1016/j.ebiom.2017.06.016

**Published:** 2017-06-19

**Authors:** Neel Heerasing, Nicholas A. Kennedy

**Affiliations:** Exeter IBD group, Royal Devon and Exeter NHS Foundation Trust, United Kingdom

Crohn's disease (CD) is a common form of chronic inflammatory bowel disease (IBD) affecting 21–294 per 100,000 in European populations (Cosnes et al., 2011). It arises most likely from an interaction between genetic and environmental factors leading to an aberrant immune response to the gut microbiota. Previous genome-wide association studies have described more than 200 genetic loci that are associated with IBD (Liu et al., 2015). *NOD2* (nucleotide-binding oligomerisation domain containing 2), an intracellular sensor of bacteria-derived muramyl dipeptide, was the first genetic association discovered in CD and remains one of the strongest associations (Knights et al., 2013). The mechanisms whereby *NOD2* mutations result in intestinal inflammation in CD remain incompletely understood. Evidence suggests that NOD2 mutations are associated with a loss of innate immune protective mechanisms in both circulating antigen presenting cells and in the intestine (Van Heel et al., 2006). The three main reported disease-associated polymorphisms (R702W, G908R, and 1007fs) all contribute to disease pathogenesis through interference with bacterial recognition (Van Heel et al., 2006).

Several environmental factors have been postulated in CD, but the most consistent epidemiological evidence points to a clear risk associated with cigarette smoking. Two meta-analyses reveal that current smokers are up to twice as likely to develop CD as non-smokers (OR = 1.8–2.0) (Mahid et al., 2006). Cigarette smoking can impair both innate and adaptive immune responses, thereby increasing susceptibility to microbial infections which would support its role in the pathogenesis of CD (Sopori, 2002).

The role of genetics and environmental contribution to the aetiology of CD has been the subject of much research over the past decade. It has been reported that cigarette smoke extract can delay *NOD2* mRNA expression leading to the impairment of NOD2 functioning in intestinal epithelial cells (Helbig et al., 2012).

Given the established roles of NOD2 and cigarette smoking on innate immunity, it is conceivable that cigarette smoking might modulate the functional consequences of CD-associated polymorphism in *NOD2* (Helbig et al., 2012).

Kuenzig et al. (2017)) describe a study of the interaction of smoking and one of three loss-of-function risk variants in the *NOD2* gene (1007fs). This is the first comprehensive systematic review and meta-analysis of the interaction between *NOD2* and cigarette smoking in CD. Whilst it is clear that smoking and *NOD2* variants increase the risk of CD independently, there is conflicting evidence for their interaction with some studies suggesting a positive interaction, a negative interaction or no interaction. The discordant findings could be explained by the fact that the majority of previous *NOD2*/smoking studies were underpowered to detect an interaction. Based on a large literature review and meta-analysis, the authors conclude that only one of the three known CD risk variants in *NOD2* (1007fs) shows an interaction with smoking exposure and more importantly, this interaction is negative. They subsequently explore this relationship in more detail in a case series of 627 patients which confirms the negative interaction. They suggest this is due to the inverse relationship between *NOD2* prevalence (increased in younger patients with CD) and smoking exposure (increased in older patients with CD) with increasing age of onset. It is important to point out that the authors could not restrict their analysis to individual age groups and hence, they were not able to adjust age as a confounder. However, a similar significant negative interaction between smoking and NOD2 was demonstrated in a previous study by Helbig et al. (2012) even after adjusting for age at diagnosis. They concluded that the risk increase for CD conferred simultaneously by cigarette smoking and the 1007fs *NOD2* polymorphism is smaller than expected under a multiplicative model.

The complexity of identifying gene-environment interactions for Crohn's disease should be acknowledged. It is important to understand the design of the studies and the methodical implications of grouping multiple genetic variants into a single analysis. Helbig et al. were the first to use a case-only design to explore statistical interaction and the current meta-analysis adopted a similar design. A multiplicative interaction as demonstrated here does not indicate which group is at a higher absolute risk from the environmental exposure; case-only studies cannot be used to assess for additive interactions, and it remains possible that the absolute risk of smoking in *NOD2* variant carriers would be higher than in those with wild-type *NOD2* (Greenland et al., 2008).

The similar results obtained from the two quoted studies are intriguing and point to the importance of assessing genetic and environmental factors in sub-phenotypes of CD. In addition to age, disease location, disease behaviour and other subphenotypes may influence gene-environment interactions. Both *NOD2* and smoking are associated with ileal CD and furthermore, ileal and colonic CD have been shown to be in part genetically-determined phenotypes (Cleynen et al., 2016).

Further epidemiological and functional studies are needed to fully explore the mechanistic gene-environment interactions in patients with CD. They need to be adequately powered to assess SNP-specific interactions. The current meta-analysis provides a platform for further research in the field of precision medicine which will enable clinicians to develop personalised recommendations for individuals at high risk of developing CD.

## Disclosure

The authors declared no conflicts of interest.
